# Combinatorial selection for replicable RNA by Qβ replicase while maintaining encoded gene function

**DOI:** 10.1371/journal.pone.0174130

**Published:** 2017-03-22

**Authors:** Mio Yumura, Natsuko Yamamoto, Katsushi Yokoyama, Hirotada Mori, Tetsuya Yomo, Norikazu Ichihashi

**Affiliations:** 1 Graduate School of Information Science and Technology, Osaka University, 1–5 Yamadaoka, Suita, Osaka, Japan; 2 Graduate School of Biological Sciences, Nara Institute of Science and Technology, Takayama, Ikoma, Japan; 3 Department of Biomedical Ethics and Public Policy, Graduate School of Medicine, Osaka University, 2–2, Yamadaoka, Suita, Osaka, Japan; 4 East China Normal University, Shanghai, P.R.China; 5 Graduate School of Frontier Biosciences, Osaka University 1–5 Yamadaoka, Suita, Osaka, Japan; Southern Illinois University School of Medicine, UNITED STATES

## Abstract

Construction of a complex artificial self-replication system is challenging in the field of in vitro synthetic biology. Recently, we developed a translation-coupled RNA replication system, wherein an artificial genomic RNA replicates with the Qβ RNA replicase gene encoded on itself. The challenge is to introduce additional genes into the RNA to develop a complex system that mimics natural living systems. However, most RNA sequence encoding genes are not replicable by the Qβ replicase owing to its requirement for strong secondary structures throughout the RNA sequence that are absent in most genes. In this study, we establish a new combinatorial selection method to find an RNA sequence with secondary structures and functional amino acid sequences of the encoded gene. We selected RNA sequences based on their in vitro replication and in vivo gene functions. First, we used the α-domain gene of β-galactosidase as a model-encoding gene, with functional selection based on blue-white screening. Through the combinatorial selection, we developed more replicable RNAs while maintaining the function of the encoded α-domain. The selected sequence improved the affinity between the minus strand RNA and Qβ replicase. Second, we established an in vivo selection method applicable to a broader range of genes by using an *Escherichia coli* strain with one of the essential genes complemented with a plasmid. We performed the combinatorial selection using an RNA encoding *serS* and obtained more replicable RNA encoding functional *serS* gene. These results suggest that combinatorial selection methods are useful for the development of RNA sequences replicable by Qβ replicase while maintaining the encoded gene function.

## Introduction

In the field of in vitro synthetic biology or artificial cell synthesis, in vitro synthesis of several types of biological functions is being attempted to understand the design principles of biological systems or to develop new technologies [1,2,3,4,5,6,7]. One of the central functions of living things is to express genetic information as functional molecules, proteins, or RNAs. This process has been reconstituted in vitro using crude cell extracts [8,9] or purified components [10], and complex gene regulatory networks have been developed using these reconstituted systems [11,12]. Another important biological function is the replication of genetic information as DNA or RNA, which has been reconstituted using only RNA molecules [13,14] or a combination of RNA, DNA, and proteins [15,16,17].

Recently, we have coupled an RNA replication system with a reconstituted gene expression system to develop a translation-coupled RNA replication system, wherein an artificial RNA genome replicates by the Qβ RNA replicase encoded on itself [18]. When we repeat the RNA replication for many generations in a micro-scale compartments, mutants that replicate faster dominate the population based on Darwinian principle [19]. One of the next important challenges is to introduce new genes into the RNA genome to complicate the replication system and to mimic natural systems. However, we found that introduction of a new gene into the RNA genome completely abolished replication by the Qβ replicase because the replicase requires strong secondary structures throughout the RNA, which are absent in most genes [20]. Simple introduction of mutations that generate strong structures does not solve this problem because mutations often change the amino acid sequence of the encoded gene and hamper gene function. Therefore, to develop a more complex RNA replication system, we established a strategy to obtain a replicable RNA while maintaining the encoded gene function.

One of the possible strategies is designing the RNA structure by using only synonymous mutations. In a previous study, we attempted this strategy and succeeded in developing an RNA sequence encoding the α-domain gene of β-galactosidase to be replicable at a certain level [20]. However, this strategy relies on RNA structure prediction algorithms, which are only accurate for short sequences and require several rounds of trial-and-error. Therefore, we needed another strategy that is applicable to larger RNAs.

In this study, we attempted to establish an evolutionary strategy to obtain a replicable RNA while maintaining the encoded gene function. Using RNAs encoding the α-domain gene or *serS* as model genes, we performed a combinatorial selection cycle, consisting of two types of selection, replication selection, in which more replicable RNAs are selected, and functional selection, in which RNAs encoding the functional genes are selected. We repeated this cycle for 10 rounds, and obtained more replicable RNAs that encode the functional genes.

## Result

### Selection cycle

**Fig 1:**
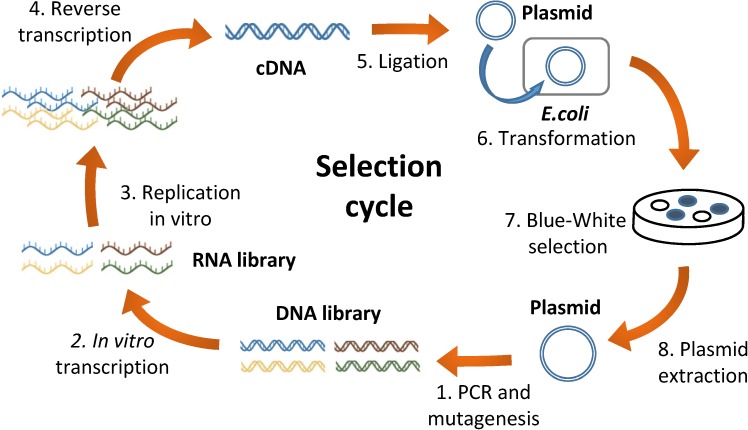
Scheme of the first selection cycle. The selection cycle consists of two types of selection: in vitro selection of more replicable RNA and in vivo functional selection of the α-domain gene of β-galactosidase. (1) DNA fragments containing the α-domain gene, the terminal recognition sites for Qβ replicase, and the T7 promoter were amplified, and mutations were introduced by error-prone PCR to obtain a DNA library. (2) The DNA library was transcribed in vitro with T7 RNA polymerase to produce the RNA library. (3) In vitro RNA replication was performed with Qβ replicase. In this step, more replicable mutant RNAs in the library were selected. (4) The replicated RNAs were reverse transcribed into cDNAs, (5) ligated to a plasmid vector and (6) transformed into an *E*. *coli* strain that expressed the ω-domain of β-galactosidase. (7) The transformed *E*. *coli* were plated on an agar plate containing the colorimetric substrate of β-galactosidase. The cells harboring the plasmids containing the functional α-domain gene produced blue-colored colonies and were collected. (8) The plasmids were then extracted from the colony mixture for the next cycle.

In this study, we attempted to establish a combinatorial selection cycle through in vivo functional selection and in vitro replication selection. As a gene encoded in RNA, we first chose the α-domain of β-galactosidase because the gene function can be easily selected on an agarose plate containing the colorimetric substrate for β-galactosidase through blue-white screening [21] based on α-complementation [22]. Fig 1 shows the scheme of the first selection system. (1) We performed error-prone PCR to amplify and mutagenize the region including the α-domain gene to generate a DNA library. (2) The library was transcribed in vitro to produce an RNA library. (3) In vitro replication was performed with the purified Qβ replicase. (4) cDNA was synthesized through reverse transcription. (5) The cDNA was ligated to a plasmid vector containing the T7 promoter. (6) The plasmid was introduced into an *Escherichia coli* strain expressing the ω-domain of β-galactosidase for α-complementation. The cells were then inoculated on an agar plate containing a colorimetric substrate for β-galactosidase and an antibiotic to select the plasmid-harboring cells. If an *E*. *coli* cell has a plasmid harboring the functional α-domain gene, it produces a blue colony (Blue-white selection [21]). (7) About 30–100 blue-colonies were picked up and pooled. (8) Plasmids were extracted from the colony mixtures. This cycle was started with an RNA sequence encoding the α-domain gene obtained in our previous study [20] and was repeated for 10 rounds.

### Clone assay

**Fig 2:**
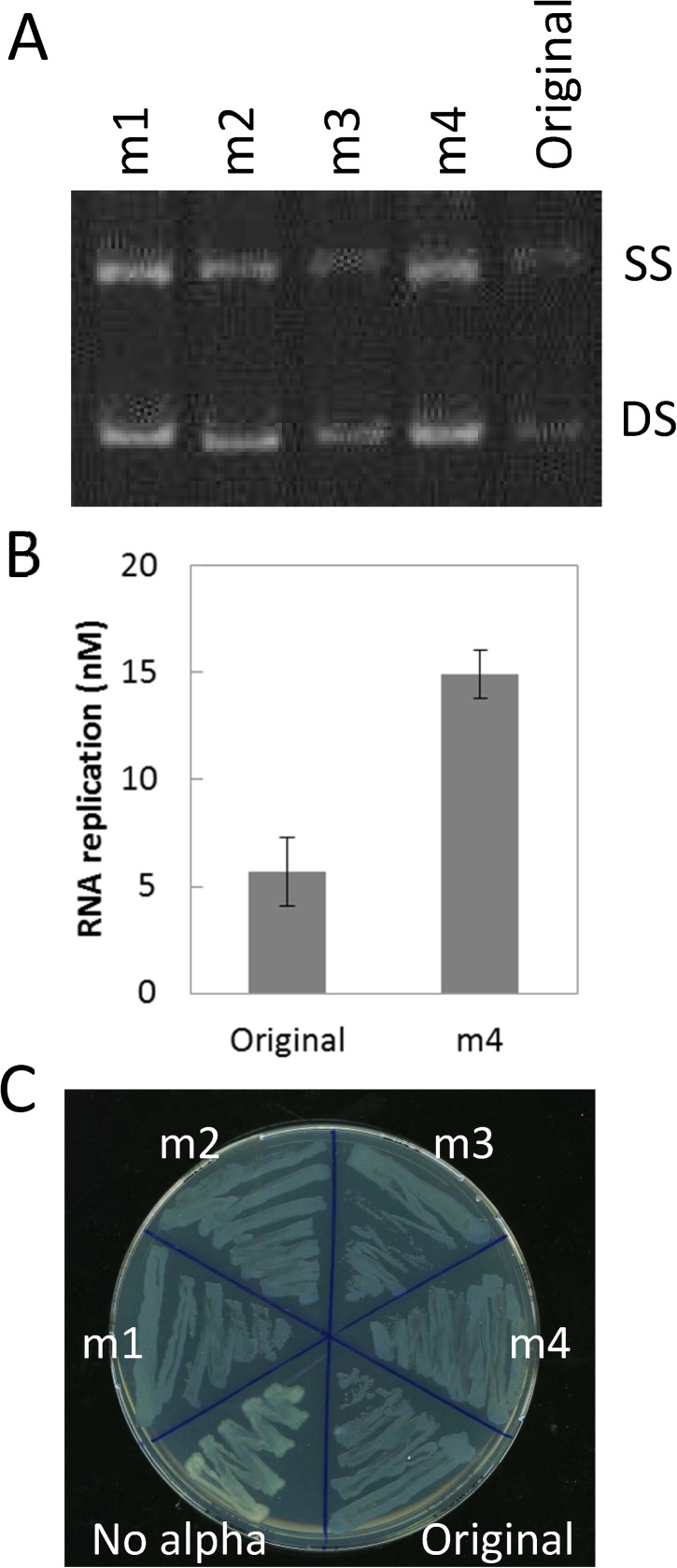
Replication and α activities of RNA clones after the selection cycle. A) Four RNA clones were randomly chosen after the selection cycle. These RNAs (0.1 nM) were replicated with the Qβ replicase (100 nM) for 1 h at 37°C and subjected to 8% polyacrylamide-gel electrophoresis followed by RNA staining with SYBR green II. SS and DS indicate the position of single- or double-strands, respectively. B) To obtain quantitative data, the RNA concentration of the original and m4 RNA in the replicated solutions were measured by quantitative PCR after reverse-transcription. Error bars represent standard error. C) The α-complementation activities of the α-domain genes. The plasmids encoding each RNA clone were introduced into an *E*. *coli* strain expressing the ω-domain gene, and the cells were streaked on a plate containing the colorimetric substrate for β-galactosidase. If the plasmids contain functional α-domain gene, the cell streaks become blue.

We randomly chose four blue colonies in the final round and extracted each plasmid clone, which was used to prepare each RNA clone (m1–m4). Sequencing analysis showed that various different mutations were introduced in these RNAs (Table 1). To examine the replication abilities of these RNAs, we performed a replication assay with 0.1 nM of each RNA and 100 nM replicase for 1 h at 37°C, and the reaction was subjected to polyacrylamide-gel electrophoresis followed by RNA staining with SYBR green II (Fig 2A). Three of the mutant RNAs (m1, 2, and 4) exhibited greater amounts of single- or double-stranded RNA (SS or DS) than the original RNA, indicating that clones with higher replicative abilities were selected through the selection cycles. To quantitatively analyze the replication, we measured the RNA concentration for m4 RNA by quantitative PCR after reverse-transcription and found that RNA was replicated by more than 2-times compared to the original RNA (Fig 2B).

**Table 1:** List of mutations.

	m1	m2	m3	m4
5'UTR				
A61G	+			
T69C			+	
T74C		+		
T80del		+	+	+
T81del	+	+	+	+
A87C	+	+	+	+
α-domain gene region				
T112C (I4T)	+			
G116T	+	+	+	+
T138A (S13T)	+	+	+	+
A146T		+		
T264C (W55R)				+
T269C	+			
A271G (N57S)	+	+	+	+
C290T	+	+	+	+
C303T (Q68stop)		+	+	
A307C (Q69P)		+	+	
T323C	+	+		
A329T (E76D)			+	
A334T (stop78L)	+	+		
3′UTR				
A336G				+

We then examined whether the clones sustained the function of α-domain gene. We introduced the plasmid clone encoding each RNA into *E*. *coli* cells expressing the ω-domain gene and streaked them on an agar plate containing the colorimetric substrate of β-galactosidase. The *E*. *coli* containing no plasmid exhibited white colored streaks, whereas those containing the original α-domain gene and m1 to m4 exhibited blue colored streaks (Fig 2C), indicating that all the selected clones maintained the functional α-domain gene.

**Fig 3:**
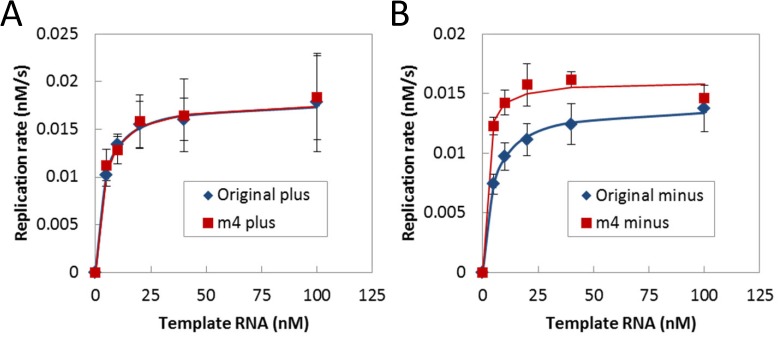
Estimation of the kinetic parameters of the original and m4 RNAs. To estimate the kinetic parameters, K_M_ and k_cat_, the rates of complementary strand synthesis were measured using the plus (A) or minus strands (B) of the original and m4 RNA clones. Each template RNA (0, 5, 10, 20, 40, and 100 nM) was incubated with the replicase (10 nM) at 37°C for 5 min, and the synthesized RNA was measured as described in the Materials and Methods section. The plots were fitted to Michaelis-Menten equations (solid lines) for estimating V_max_ and K_M_. To estimate the k_cat_ values, we used the active ratio of the replicase (20%) [23]. The estimated parameters are shown in Table 2 along with the fitting errors. The error bars represent standard errors (n = 3).

We then analyzed one of the clones, m4, in more detail to understand how the introduced mutation improved the replication activity. RNA replication consists of two RNA polymerization events, complementary minus strand synthesis from the initial template plus strand, which encodes the α-domain gene, and plus strand synthesis from the minus strand. To analyze both events, we prepared the minus strand RNA and measured the kinetic parameters for both the plus and minus strands. We performed RNA replication with various initial concentrations of plus or minus RNA for the original and the m4 RNA and then measured the replication rates. The plots were fitted to a Michaelis-Menten curve to estimate the two parameters, K_M_ and k_cat_ (Fig 3 and Table 2). The k_cat_ values were not significantly different between the original and m4 RNAs for both the plus and minus strands. The K_M_ values for the original and the m4 plus strands were also similar, whereas the K_M_ value of the m4 minus strand was approximately 3-times smaller than that of the original minus strand. This indicates that improvement of replication for m4 RNA is primarily attributed to the decreased K_M_ value of the minus strand.

**Table 2:** Kinetic parameters.

	*K*_*M*_(nM)	*k*_*cat*_(1/s)
original plus	3.8 ± 0.4	0.018 ± 0.0004
m4 plus	3.6 ± 0.6	0.018 ± 0.0006
original minus	4.6 ± 0.4	0.014 ± 0.0003
m4 minus	1.3 ± 0.6	0.016 ± 0.0007

**Fig 4:**
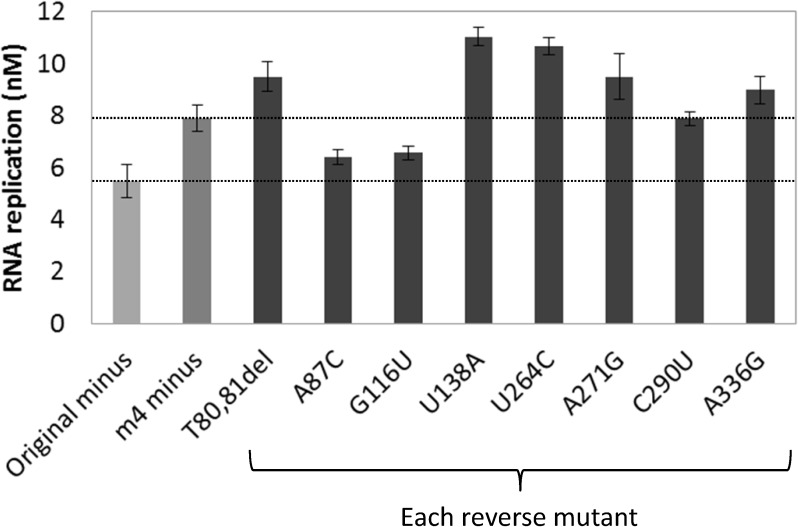
RNA replication of reverse mutations from the m4 minus RNA. Eight reverse mutant RNAs (10 nM), in which each of the eight mutations introduced in the m4 minus RNA were reverted to the original nucleotides, were incubated with the replicase (10 nM) for 0.5 h at 37°C. The replication amount was measured as described in the Materials and Methods. For comparison, the replication level of the original and m4 minus RNA are shown (dotted lines). The error bars represent standard errors (n = 3).

We then attempted to identify the mutations responsible for increasing the replication ability of the m4 minus strand. The m4 mutant has eight mutations including seven point mutations and one deletion. We prepared eight RNAs in which each of the eight mutations was reversed to the original sequence and then we performed replication with these reverted RNAs (Fig 4). When the mutations, A87C and G116U, were reverted, the replication amounts were decreased to the level of the original RNA, indicating that these two mutations are responsible for the improved replication ability of m4. Unexpectedly, other reverse mutations including the 80-81UU deletion, U138A, U264C, A271G, and A336G, showed higher replication amounts than the m4 mutant, indicating that these mutations are inhibitory to replication.

**Fig 5:**
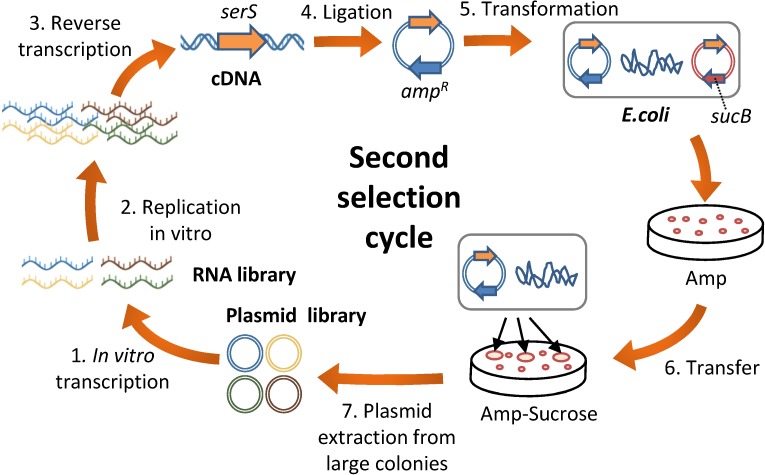
Scheme of the second selection cycle. The second selection cycle is the same as the first one (Fig 1) except for the changes in the in vivo selection method and omission of PCR amplification and mutagenesis. (1) A plasmid encoding the target gene, terminal recognition sites for Qβ replicase, and T7 promoter was transcribed in vitro with T7 RNA polymerase to produce an RNA. (2) In vitro RNA replication was performed with Qβ replicase. In this step, more replicable mutant RNAs from the library were selected. (3) The replicated RNAs were reverse transcribed into cDNAs and (4) ligated to a plasmid vector. (5) The ampicillin resistance plasmid was introduced into an *E*. *coli* strain with deleted target gene and complemented with a plasmid encoding *sucB* gene for negative selection. (6) After 10h incubation in the presence of ampicillin, the small colonies were transferred on a new agar plate containing 5% sucrose for selection of cells that lost the original complementary plasmid. An *E*. *coli* cell harboring the plasmid encoding the functional target gene would produce a large colony on this plate. (7) All large colonies were collected and plasmids extracted from the colony mixtures for the next cycle.

We next attempted to apply the combinatorial selection method to other genes. Given that the present method employing blue-white selection is not applicable to other genes, we developed an alternative in vivo selection method, as described in Fig 5. (1) The plasmid library encoding a target gene was transcribed in vitro to produce the RNA library. (2) In vitro replication was performed with the purified Qβ replicase. (3) cDNA was synthesized through reverse transcription. (4) The cDNA was ligated to a plasmid vector containing the T7 promoter and ampicillin resistance gene. (5) The plasmid was introduced into an *E*. *coli* strain with deleted target gene and complemented with a plasmid encoding *sucB* gene for negative selection. The cells were streaked on an agar plate containing ampicillin for the selection of cells harboring the introduced plasmid. (6) After 10-h incubation, the small colonies were transferred on to a new agar plate containing 5% sucrose for selection of cells that lost the original complementary plasmid. An *E*. *coli* cell harboring the plasmid encoding the functional target gene would produce a large colony on this plate. All large colonies were picked up and pooled. (7) Plasmids were extracted from the colony mixtures.

**Fig 6:**
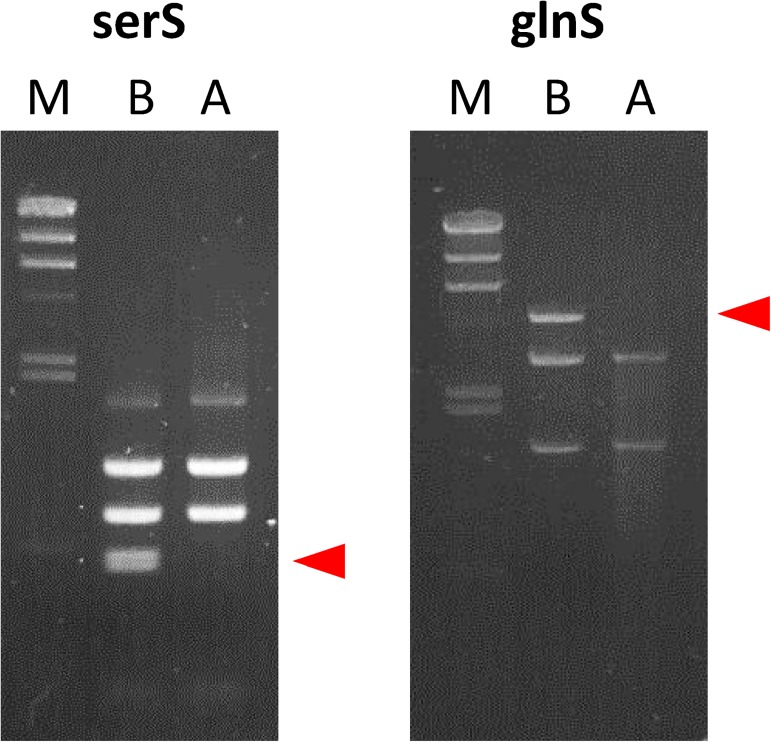
Model selection experiments of *serS* and *glnS*. A 1:1 mixture of plasmids encoding wild-type gene and non-functional mutant gene was subjected to in vivo functional selection (steps [5–7] in Fig 5). The plasmid mixture before (lane B) and after (lane A) selection was directly digested with *Eco*RV and *Pst*I for *glnS* or digested with *Cla*I after PCR amplification with primers, GGCGATTAAGTTGGGTAACGCCAG and CCGGCTCGTATGTTGTGTGG for *SerS*. Bands specific for mutant gene were indicated with arrowheads. The “M” lane indicates λ-*Hind* III marker.

To test the applicability of the second selection cycle for selection of a functional gene, we first performed model selection experiments for two genes, *serS* and *glnS*. The plasmid encoding each gene was mixed with the same amount of the plasmid encoding the non-functional mutant counterpart and applied to in vivo functional selection (steps [5–7] in Fig 5). The plasmid mixtures before and after selection were digested with restriction enzymes to distinguish between the wild-type and non-functional mutant genes. The non-functional mutant genes showed specific bands (Fig 6, arrowheads). For both *serS* and *glnS*, mutant-specific bands were observed before selection (lane B) but disappeared after selection (lane A). Thus, we demonstrated that functional gene is selected using the in vivo selection method.

**Fig 7:**
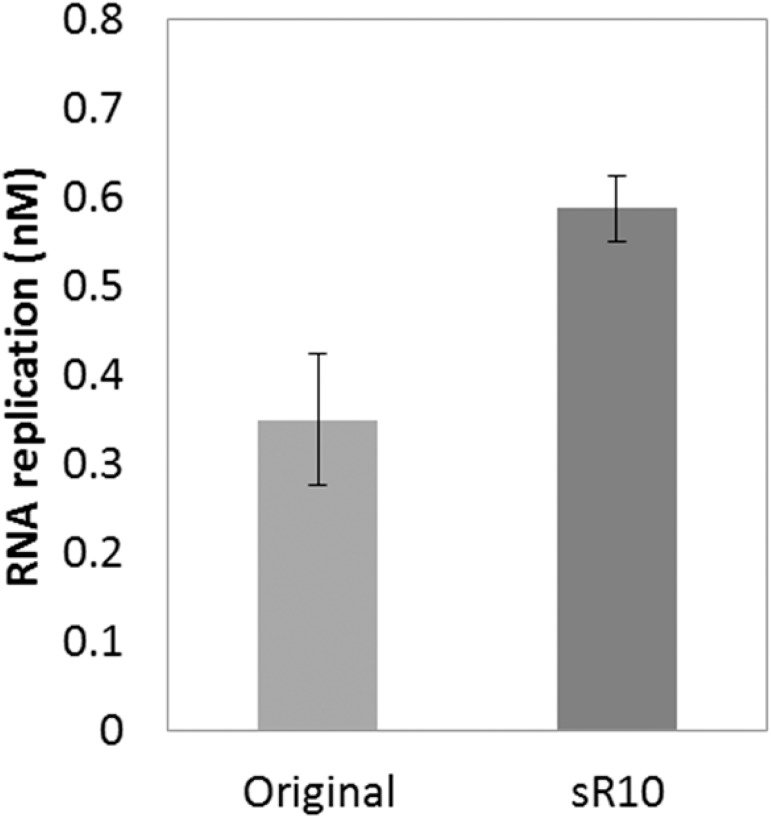
Replication of a *serS* RNA clone after the combinatorial selection cycle. The original RNA and the RNA (sR10) obtained after the combinatorial selection were incubated with replicase (20 nM) for 0.5 h at 37°C. The replication amount was measured as described under Materials and Methods. Error bars represent standard errors (n = 3).

We further attempted to develop more replicable RNA that encodes functional *serS* through the selection cycle. We repeated the cycle for 10 rounds and extracted plasmids from four independent large colonies. Only one plasmid showed mutations in the transcribed region, T779C and A1121G, both of which are in *serS* coding region but do not change the amino acids sequence. The replication ability of the mutant *serS* RNA (sR10) was approximately 1.7-fold higher than that of the original RNA before selection cycle (Fig 7). Thus, we conclude that through the combinatorial selection cycle, we obtained more replicable RNA that encodes functional *serS* gene.

## Discussion

We performed a combinatorial cycle of in vitro replication selection and in vivo functional selection using blue-white selection to obtain four mutant RNAs encoding the α-domain gene of β-galactosidase. Three of these four mutant RNAs showed replication ability significantly higher than that of the original RNA, while maintaining the encoded α-domain gene function. Kinetic analysis of one clone (m4) revealed that the affinity between its minus strand and Qβ replicase was improved attributed to at least two point mutations, A87C and G116U. In addition, we developed another type of combinatorial cycle applicable to a broader range of genes and obtained more replicable RNA encoding functional *serS* gene. Taken together, these results show that the combinatorial selection strategy is useful for the development of RNA sequences replicable by Qβ replicase while maintaining the encoded gene function.

The first combinatorial selection cycle (Fig 1) relies on blue-white selection and therefore applicable only to genes with a colorimetric substrate. We overcame this limitation with the design of the second combinatorial selection cycle (Fig 5) potentially applicable to all the essential genes of *E*. *coli*. This method would contribute to obtain replicable RNAs that encode various gene types. Such replicable RNAs would allow the development of more complex RNA self-replication systems that would mimic the natural living systems.

The mechanism of how the mutations introduced in the selection cycle increased RNA replication is presently unknown. Our previous studies show the importance of strong RNA structures throughout the template RNA, given that in their absence the newly synthesized RNA strand hybridizes with the template RNA strand to form double-stranded RNA—a dead-end product for RNA replication [20,24,25]. Although we have analyzed structures of the mutant RNA sequence obtained in this study, no clear pattern of structure change was observed. Thus, there must be other parameters, aside from strong RNA structure, that qualify an RNA to be a good template for Qβ replicase.

The reverse mutant RNAs of 80-81UU deletion, U138A, U264C, A271G, and A336G unexpectedly exhibited higher replication ability as compared to the original m4 mutant (Fig 4). Hence, these mutations are inhibitory to replication, even though they are introduced during the selection cycle. One possible reason is the interaction between mutations; these mutations were not inhibitory when first introduced into the RNA but become inhibitory after some other mutations were introduced. Another plausible reason may be the high mutation rate in this study. We introduced mutations with error-prone PCR at every round, which could introduce multiple mutations at one time. Therefore, some of the deleterious mutations could be introduced together with a few beneficial mutations and become fixed in the population by hitchhiking. Such multiple mutations might have decreased the selection efficiency. Therefore, we omitted the mutagenesis process in the second selection cycle (Fig 5) and found that mutations were introduced even without mutagenesis, probably owing to the error-prone RNA replication with Qβ replicase. The mutation rate during RNA replication is estimated to be approximately 1.5×10^−3^ per base per replication [26], corresponding to 1–2 mutations per *serS* gene (1.3 kbases). This number (approximately one mutation per gene) is considered to be appropriate for directed evolution [27]. This result indicates that mutagenesis process should be omitted for the selection of genes more than 1 kbase.

## Materials and methods

### RNA preparation

The original RNA encoding α-domain gene was synthesized by in vitro transcription with T7 RNA polymerase from the plasmid, pUC-mdv-alpha180-mutL2, produced by removing the 3′-terminus α-domain gene until 180 nt from the plasmid, pUC-mdv-alpha-m14, in our previous study [20]. The 3′ terminus was removed because it was known to increase the α-complementation activity [28]. The other RNAs, m1 –m4, were generated from each corresponding plasmid, pUCda2-mdv-alpha180-mut1-4. The RNA encoding serS and glnS were transcribed from plasmids, pUC-mdv-SerRS, pUC-mdv-glnS, respectively. These plasmids were constructed by ligating DNA fragments containing serS or glnS genes, PCR amplified using the each expression plasmids [10] as template and primers, 5’-GGAGATATACACATGCTCGATCCCAATCTGCTG and 5’-GCGGCCGCAAGCCTAGCCAATATATTCCAGTCCGTTC, with vector DNA fragments, PCR amplified using the R128 clone in the previous study [19] as template and primers 5’-TAGGCTTGCGGCCGCAC and 5’-CATGTGTATATCTCCTTCTTAGAGTTAAAC. To construct the plasmids encoding non-functional serS and glnS, frameshift mutations were introduced by PCR using primers GAAGATCGATCGAGCCTTTACGTCTGGAAG and GCTCGATCGATCTTCCCCGCGCGCTTTC for serS or AAGATCGATCGAGTATGTTGAGTCGATCAAAAAC and TACTCGATCGATCTTCTTTTACCGGGTTAGTGTC for glnS. These frameshift mutations erased the first EvoRV sites and produces ClaI sites.

### First combinatorial selection cycle

The original RNA (0.1 nM) was replicated using Qβ replicase (100 nM) purified as described previously [29] in the replication buffer, 125 mM Tris-HCl (pH 7.8), 5 mM MgCl_2_, 0.005% bovine serum albumin, 1.25 mM each NTP, and 1.25 units/μl RNase Inhibitor (Promega) at 37°C for 1 h. The replicated RNA was reverse-transcribed at 50°C for 30 min with PrimeScript reverse transcriptase (Takara, Japan) using the RT primer, 5’-CGTACGGGAGTTCGACCGTG, according the manufacturer’s instruction. The cDNA was PCR amplified with KOD FX DNA polymerase (Toyobo, Japan) using RT primers and another primer, 5’-CGAAAGCGCTAGCCCGTGAC. The PCR product was purified with the PureLink DNA purification kit (Thermo Fisher Scientific) and was ligated to a vector fragment by using the In-Fusion HD cloning kit (Takara, Japan) according the manufacturer’s instructions. The vector fragment was prepared by PCR using the plasmid, pUCda2-mdv-alpha(+) from which the α-domain gene originated in the pUC vector was removed, as the template and primers, 5’-AGTCACGGGCTAGCGCTTTC and 5’-TCGAACTCCCGTACGAGGTGCC. The product was transformed into an *E*. *coli* strain (JM109) and spread on an agar plate containing 50 μg/ml ampicillin, 0.5 mM isopropyl β-d-1-thiogalactopyranoside, and 40 μg/ml Bluo-Gal (Thermo Fisher Scientific). After more than 24 h of incubation, 30–100 colonies were collected and pooled. Plasmids were extracted from the colony mixture by using the PureLink plasmid miniprep kit (Thermo Fisher Scientific). Using these extracted plasmids as templates, mutations were introduced by error-prone PCR using the primers, 5’-CGCAACGCAATTAATGTGAG and 5’-GCGTCAGCGGGTGTTGG, with slight modifications from Cadwell et al [30]. The error-prone PCR solution contained 10 mM Tris-Cl (pH 8.3), 50 mM KCl, 7 mM MgCl_2_, 1 mM dCTP, 1 mM dTTP, 0.2 mM dATP, 0.2 mM dGTP, and 2 μM each primers, 0.5 mM MnCl_2_, 0.05 unit/μl Taq DNA polymerase, and 160 pg/μl template plasmids. The PCR product was then digested with SmaI and transcribed to RNA with T7 RNA polymerase. The RNA was purified with the PureLink RNA purification kit (Thermo Fisher Scientific) and was used for the next round of replication.

### RNA replication

In the experiment shown in Fig 2A, RNA replication was performed by the same method as that in the selection cycle, and the reaction mixture was then subjected to 8% polyacrylamide-gel electrophoresis followed by staining with SYBR green II (Takara, Japan). In the experiment shown in Figs 3 and 4, the replication solution contained 25 mM Tris-HCl (pH 7.8), 5 mM MgCl_2_, 0.005% bovine serum albumin, 1.25 mM each NTP, and 1.25 units/μl RNase Inhibitor (Promega), 10 nM Qβ replicase, the indicated amount of template RNA, and 15 kBq/μl [^32^P]-UTP. The solution was incubated for 5 min (Fig 3) or 30 min (Fig 4 and Fig 7) at 37°C, and subjected to 8% polyacrylamide-gel electrophoresis with 0.1% sodium dodecylsulfate (SDS) in TBE buffer (pH 8.4) containing Tris(hydroxymethyl)aminomethane (100 mM), boric acid (90 mM), and EDTA (1 mM), followed by fixation in 7% acetic acids for 10 min and autoradiography. The method for the quantitative PCR after reverse-transcription performed in Fig 2B was the same as that described previously except for the primer sequences[19]. In this study, we used the primers AGCGCTAGCCCGTGACT and CCTCTTCGCTACTACGCCG for reverse-transcription and the primers, AAACAGCTATGACGATGATTACG and GGAAACAGCTATGACGATGATTACT, with each RT primer in quantitative PCR for the original and m4 RNA, respectively.

### Strains

Single gene deletion strains of essential genes, JW0876 (serS, ECK0884) and JW0666 (glnS, ECK0668), were constructed under the complementation condition as follows. First, we constructed the low copy plasmid vector, pFE604, a derivative of F plasmid as shown in S1 Fig. The vector has two *Sfi*I recognition sites that have different cohesive ends and allowed to uni-directional cloning with the *Sfi*I fragments from ASKA plasmid clones carring *serS* and *glnS* essential genes. Under the complementation from the plasmids with 0.1 mM IPTG in the medium, we removed the essential *serS* and *glnS* genes from the chromosome by λRED recombination method [31]. The primer sequences are shown in S1 Table. The pKD13 was used as the template plasmid for the resistant cassette.

We then introduced *sucB* gene into the complementary plasmid by ligating the fragment carrying the *sucB* gene with vector fragments using In-Fusion cloning kit (Takara, Japan). The *sucB* gene fragment was amplified from plasmid pCDSSara [32] by PCR with primers GGAAGCTAAAGCGGAGCCTATGGAAAAACG and AACTGCCTTAGGATATCGGCATTTTCTTTTGC, while the vector fragments were amplified from each complementary plasmid with primers TATCCTAAGGCAGTTATTGGTGCCCTTAAAC and TCCGCTTTAGCTTCCTTAGCTCCTGAAAATC.

### Second selection cycle

Steps (1–4) and (7) were the same as the first selection cycle described above except for the omission of PCR amplification and mutagenesis. The RNA was transcribed directly from plasmid. The ampicillin resistance plasmid encoding *serS* gene was introduced into an *E*. *coli* strain JW0876 by the standard heat-shock method and streaked on to a Luria-Bertani (LB)-agar plate containing 50 μg/ml ampicillin and covered with nitrocellulose filter. After 10-h incubation at 37°C, small colonies appeared on the filter. The filter together with the small colonies was transferred on to a new LB-agar plate containing 5% sucrose and incubated at 37°C for another 12–24 h. An *E*. *coli* cell harboring the plasmid carrying the functional target gene and without the original complementary plasmid would produce a large colony on this plate. All large colonies were collected.

## Supporting information

S1 Fig Construction of essential gene complementing plasmid. (PDF)

S1 Table Primer sequences used for strain construction. (PDF)
