# Supplementary material for: Combinatorial selection for replicable RNA by Qβ replicase while maintaining encoded gene function
Source: PLoS One. 2017 Mar 22;12(3):e0174130. doi: 10.1371/journal.pone.0174130 (PMC5362092; doi:10.1371/journal.pone.0174130)
Supplement: S1 Table — (PDF) [file pone.0174130.s002.pdf]

genes	terminus	Primer sequence
glnS	N-ter	5’-TAAGATCATACGCCGTTATACGTTGTTTACGCTTTGAGGAATCCACGATG***ATTCCGGGGATCCGTCGACC***
	C-ter	5’-CGCCCCATCCGGCATAGCGAAACTTAAAATTACTCGCCTACTTTCGCCCA***TGTAGGCTGGAGCTGCTTCG***
serS	N-ter	5’-AGGCTGTGGCCACATCTCCCATTTAATTCGATAAGCACAGGATAAGCATG***ATTCCGGGGATCCGTCGACC***
	C-ter	5’-GCGCTTTTTAGATTCAGAAAAATTGGGTATTAGCCAATATATTCCAGTCC***TGTAGGCTGGAGCTGCTTCG***

Table S1.   Primer sequences used for strain construction

Sequences in (blue in bold and italic) are the sequences for pKD13. The rest of sequences are for the target genes’ N and C-terminus regions for homologous recombination.

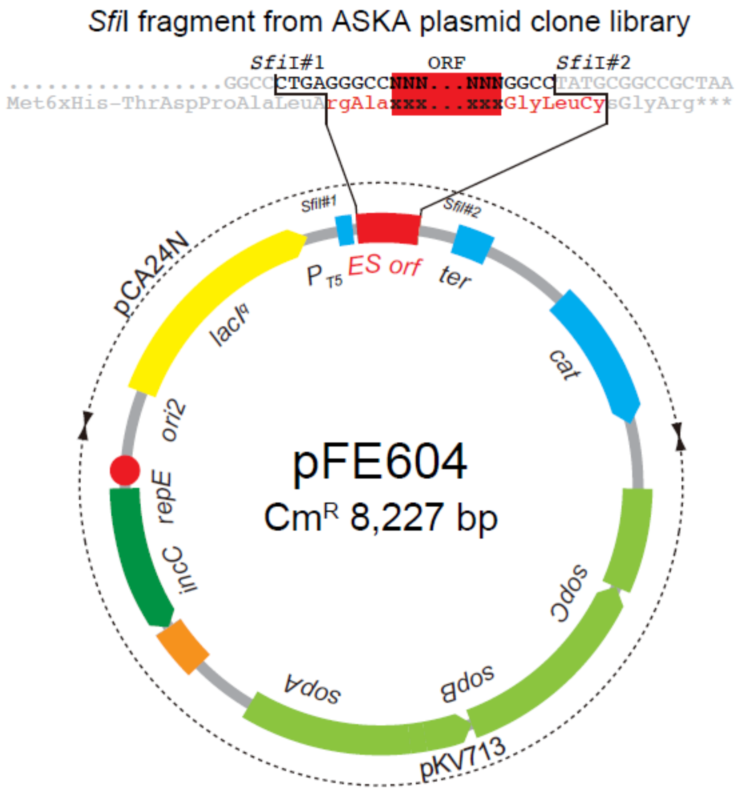

Figure S1.　Construction of essential gene complementing plasmid.

pFE604 is a derivative of F plasmid almost the same as pFE604T as previously shown [1]. The essential genes' fragments were prepared by SfiI cut of the corresponding ASKA plasmid clones [2]. pCA24N is the vector of the ASKA plasmid clone library. pKV713 is the rreplcation and partition responsibble framgnet of miniF plasmid [3]

References

1. Yong HT, Yamamoto N, Takeuchi R, Hsieh YJ, Conrad TM, Datsenko KA, Nakayashiki T, Wanner BL, Mori H: **Development of a system for discovery of genetic interactions for essential genes in Escherichia coli K-12**. *Genes Genet Syst* 2013, **88**(4):233-240. PMID: 24463526

2. Kitagawa M, Ara T, Arifuzzaman M, Ioka-Nakamichi T, Inamoto E, Toyonaga H, Mori H: Complete set of ORF clones of Escherichia coli ASKA library (a complete set of E. coli K-12 ORF archive): unique resources for biological research. DNA Res 2005, 12(5):291-299, PMID: 16769691

3. Kawasaki Y, Wada C, Yura T: Roles of Escherichia coli heat shock proteins DnaK, DnaJ and GrpE in mini-F plasmid replication. Mol Gen Genet 1990, 220(2):277-282. PMID: 2183004)
